# Rational cell culture optimization enhances experimental reproducibility in cancer cells

**DOI:** 10.1038/s41598-018-21050-4

**Published:** 2018-02-14

**Authors:** Marina Wright Muelas, Fernando Ortega, Rainer Breitling, Claus Bendtsen, Hans V. Westerhoff

**Affiliations:** 1 Manchester Centre for Integrative Systems Biology and Doctoral Training Centre, Manchester Institute of Biotechnology, University of Manchester, 131 Princess Street, Manchester, M1 7DN UK; 2 Quantitative Biology, Discovery Sciences, IMED Biotech Unit, AstraZeneca, Cambridge, UK; 3 Netherlands Institute for Systems Biology, VU University Amsterdam and University of Amsterdam, Amsterdam, The Netherlands; 4 Manchester Pharmacy School, University of Manchester, Stopford Building, Oxford Road, Manchester, M13 9PT UK; 5 Manchester Institute of Biotechnology, School of Chemistry, Faculty of Science and Engineering, University of Manchester, 131 Princess Street, Manchester, M1 7DN UK

## Abstract

Optimization of experimental conditions is critical in ensuring robust experimental reproducibility. Through detailed metabolomic analysis we found that cell culture conditions significantly impacted on glutaminase (GLS1) sensitivity resulting in variable sensitivity and irreproducibility in data. Baseline metabolite profiling highlighted that untreated cells underwent significant changes in metabolic status. Both the extracellular levels of glutamine and lactate and the intracellular levels of multiple metabolites changed drastically during the assay. We show that these changes compromise the robustness of the assay and make it difficult to reproduce. We discuss the implications of the cells’ metabolic environment when studying the effects of perturbations to cell function by any type of inhibitor. We then devised ‘metabolically rationalized standard’ assay conditions, in which glutaminase-1 inhibition reduced glutamine metabolism differently in both cell lines assayed, and decreased the proliferation of one of them. The adoption of optimized conditions such as the ones described here should lead to an improvement in reproducibility and help eliminate false negatives as well as false positives in these assays.

## Introduction

Reproducibility has increasingly become a topic of concern in biomedical research^1,2^. Scientists acknowledge that they fail to reproduce even their own experiments, let alone those of their colleagues around the globe^3^. When testing a potential anticancer drug, a novel and potent allosteric inhibitor specific for the glutaminase-1 enzyme (EC 3.5.1.2), we initially experienced a similar irreproducibility. Our focus on metabolomics led us to experiments that then produced an explanation for the lack of reproducibility, and employed a more comprehensive assay development approach which we believe can be of benefit for the scientific community. Indeed, as we go on to discuss, the use of a GLS1 inhibitor is less important here than the notion that culture conditions require optimization to minimize variability in the metabolic state of cells and to ensure normal growth of these during any assay to provide reproducible and meaningful results.

One of the initial steps in the development of therapeutic agents for cancer involves testing these agents *in vitro* using human cancer cell lines as experimental models^4,5^. Using primary cell lines in culture, the effects of compounds or perturbations on cell proliferation, DNA replication or cell death is generally investigated over a period of time. These types of read-out are highly dependent on cell physiology and as such these assays need to fulfill a number of conflicting conditions. On the one hand, cells need to be kept in culture long enough to attain a steady state and for the effects of treatments to be observed. On the other hand, they should not be kept there too long because of the gradual accumulation of waste products that can be inhibitory or toxic to cells, such as lactate and ammonia^6,7^. The concentration of nutrients will fall over time, pH will change, and as cells grow and divide, space may become limiting. As cell density increases, effects of paracrine signaling become more pronounced and as cells reach confluence, contact inhibition may suppress proliferation. Although cancer cells are able to proliferate for some time after reaching confluence by then accumulating on top of one another, this crowding still limits individual cells’ access to nutrients and growth factors^8^, eventually resulting in cell cycle arrest and apoptosis, but long before then, in shifts in cell metabolism. Cell viability assays are affected by the metabolic state of the cells and therefore any shift in metabolic states during the assay, and particularly different shifts between sensitive and resistant cell lines, would confound the outcome of such assays.

Recently, Haibe-Kains *et al*. highlighted multiple inconsistencies between two large-scale pharmacogenomic studies, the Cancer Genome Project (CGP^9^) and the Cancer Cell Line Encyclopedia (CCLE^10^), *viz*. the sensitivity profiles of common cell lines and drugs^11^. It has been suggested that differences in the cell culture conditions were amongst the reasons for these discrepancies^12,13^ and that consistency should be achievable with appropriate laboratory and analysis protocols^13^. For example, for each cell line the CGP study determined the seeding density that ensured that each was still in the growth phase at the end of the assay (~70% confluence), whilst the seeding density was not reported for the CCLE study. In addition, for adherent cells, the test compound was added ‘around 12–24 hours’ after seeding cells and studied over a further 72–84 hours in the CCLE study, whereas in the CGP study this was added 1 day after seeding and assayed 72 hours after treatment. This lack of standardized and well-described culture conditions is common to most literature in this field (see refs^14–20^ for examples). Living cells are complex; they adjust to altering environments, by quick metabolic or somewhat slower gene-expression regulation and this may readily change the extent to which any target limits cell physiology and survival. This can make results of drug targeting studies irreproducible unless the relevant environmental conditions are well controlled at the appropriate time scale. Both academia and the pharmaceutical industry recognize the necessity of much more thorough standardization to improve reproducibility^21^.

The metabolic performance of the cell lines during drug targeting assays is not assessed routinely, or at least not reported. Metabolic changes could have strong implications for therapeutic targets in, or affected by, intermediary metabolism. Metabolic enzymes involved in cellular proliferation and growth have been identified as altered in cancers, either through the expression of cancer-specific isoforms, through mutations, or through altered expression levels^22^. And it is precisely these targets that are witnessing revived interest of late^23,24^: these altered metabolic pathways are now being targeted directly, used to enhance the efficacy of existing therapeutic agents or to overcome resistance to current treatment strategies for cancer. In addition, anti-cancer drugs that do not target metabolism itself are often assayed in survival based assays. If metabolism is so involved in cell survival, its variability during survival based assays could therefore be a prime cause of irreproducibility of the outcome of the many experimental assays. We thus investigated whether variability in cellular metabolic status is linked with different phenotypic responses.

Here we show how culture conditions widely used to investigate the effects of an inhibitor of the glutaminase-1 enzyme on cell proliferation and metabolism, result in drastic and rapid changes in the metabolic state of the cells, compromising the robustness and reproducibility of the results. We then present the pipeline we engage in such cases in order to identify these changes and to optimize culture conditions accordingly. The reward is a robust study of the effects of a potentially important anti-proliferative agent.

## Results

To investigate the effect of an inhibitor (GLS1i) of the glutaminase-1 enzyme (GLS1, EC 3.5.1.2) on cell metabolism and proliferation, we started by employing culture conditions that are widely used in the scientific literature for proliferation assays and should enable the application of metabolomics^14–20^. We seeded cells at a density of 8 × 10^5^ cells/well in 1 mL of culture media 24 hours prior to commencing the experiment at a time point denoted as time 0 by adding 1.0 μM of a GLS1 inhibitor (see Materials and Methods). The effect of this inhibitor on cell survival was determined 48 hours later. We used two cell lines, A549 and H358, that are dependent on glutamine for proliferation^25^, but differ in sensitivity to a novel and potent inhibitor of GLS1 activity developed jointly by AstraZeneca and Cancer Research Technology: proliferation of A549 cells is inhibited by this ‘GLS1i’, whereas proliferation of H358 cells is insensitive to GLS1 inhibition (Supplementary Figure S1).

### The problem: the inhibitor does not seem to work

**Figure 1:**
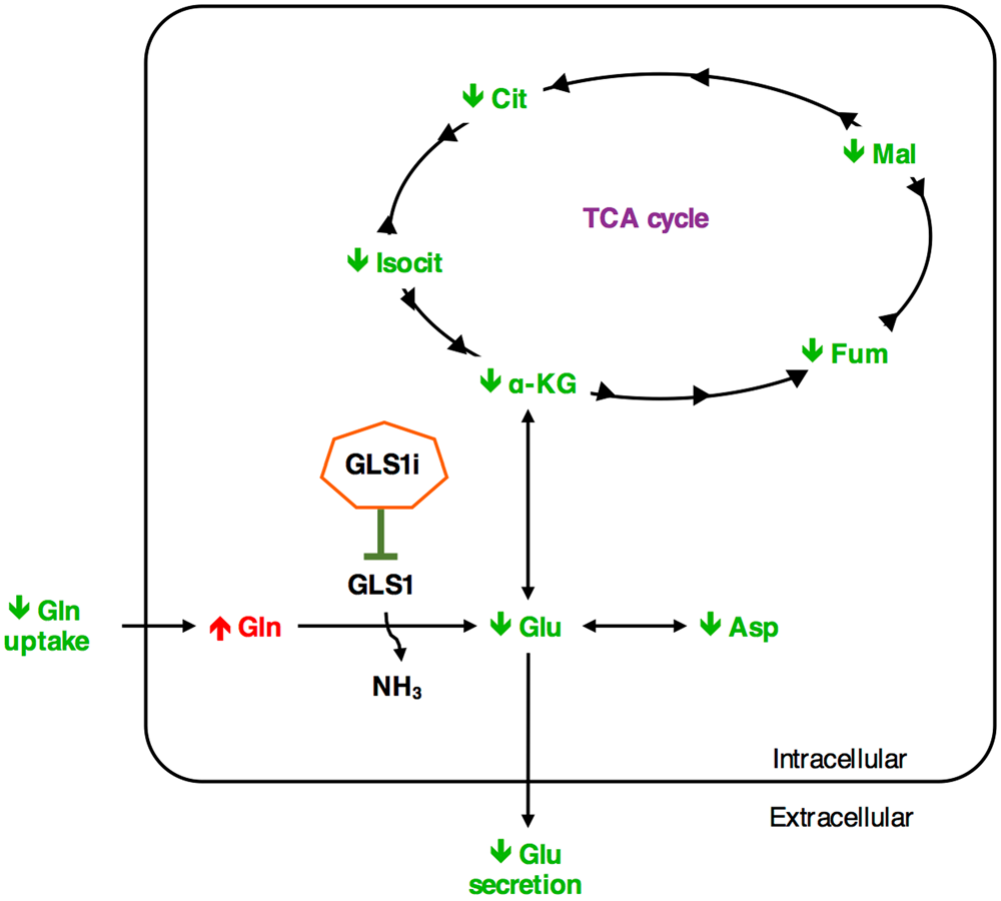
Schematic representation of the expected effects of GLS1 inhibition on metabolites proximal to the target. Shown are the key metabolites proximal to GLS1 where changes were expected following treatment with an inhibitor of this enzyme. Extracellularly, a reduction in glutamine (Gln) uptake and glutamate (Glu) secretion was expected as inhibition of GLS1 reduces the intracellular consumption of glutamine, leading to an increase in its concentration. This is followed by a reduction in cellular glutamate (Glu) and aspartate (Asp), as well as TCA cycle intermediates. This has pleiotropic effects on cells by impacting pathways that support metabolic functions needed for cell survival, growth and proliferation. α-KG, α-ketoglutarate; Cit, citrate; Fum, fumarate; Isocit, isocitrate; Mal, malate.

**Figure 2:**
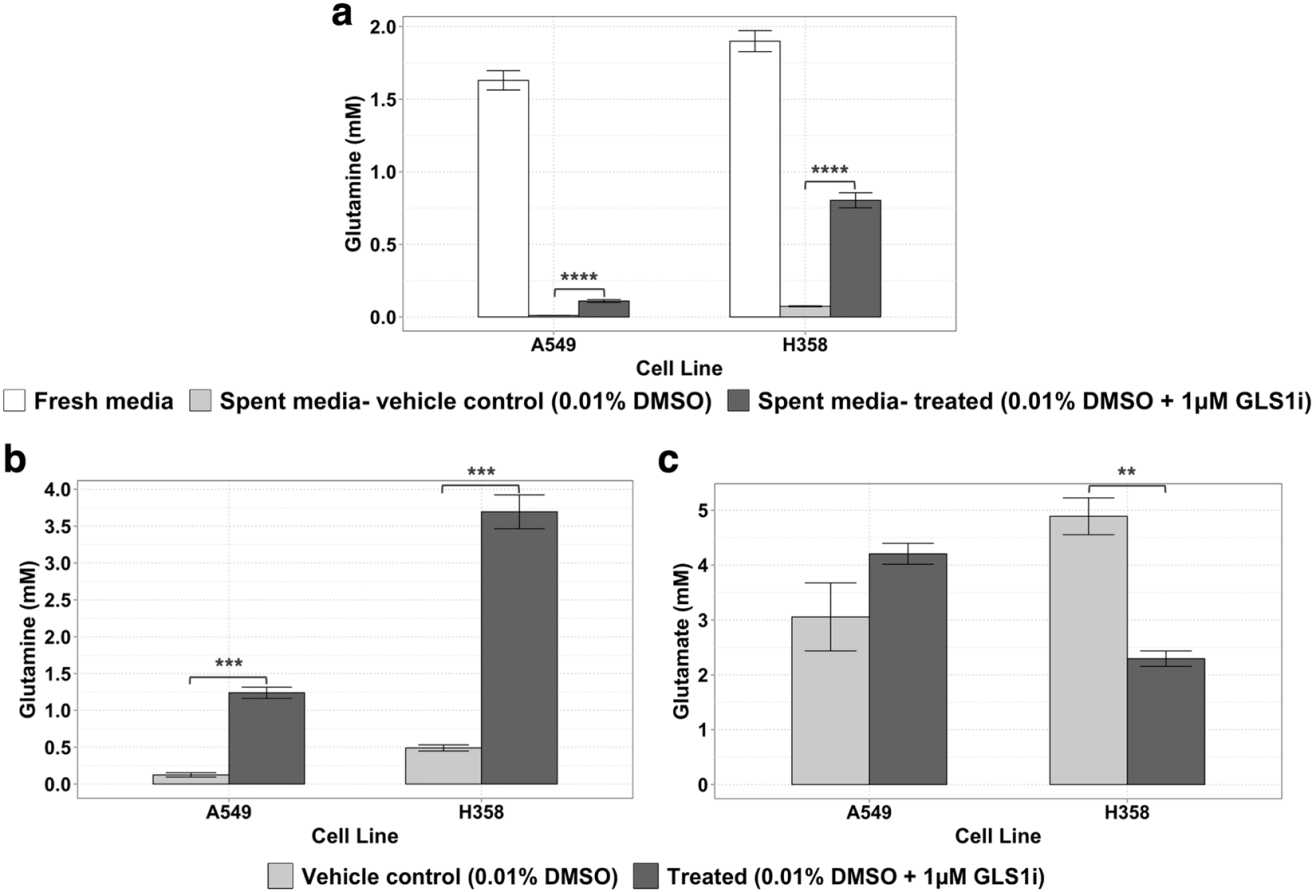
Glutamine and glutamate concentrations 48 hours after treatment with 0.01% DMSO ± 1 μM GLS1i using a prevalent assay method. A549 and H358 are known as sensitive and resistant cell lines, respectively, vis-à-vis glutaminase 1 inhibitors. Concentrations of (**a**) extracellular glutamine (**b**) intracellular glutamine and (**c**) intracellular glutamate measured by LC-UV after 24 hours of treatment with 0.010% DMSO ± 1.0 μM (final concentrations) GLS1i. For this single experiment, measurements were performed in triplicate for control and treated conditions. Cells had been seeded at a density of 8 × 10^5^ cells/well in 1 ml of culture media 24 hours prior to commencing the experiment. Shown are the mean ± SEM for the 3 technical replicates per cell line and treatment condition. Unadjusted p-values of the differences between control and treated samples obtained using a two-tailed Student’s t-test are denoted with asterisks: **p ≤ 0.01; ***p ≤ 0.001; ****p ≤ 0.0001.

We had expected that treatment with a GLS1 inhibitor would lead to a reduced consumption of glutamine, a reduced production of glutamate, an increased intracellular concentration of glutamine, a reduced intracellular concentration of glutamate and reduced intracellular concentrations of all TCA cycle intermediates (Fig. 1), particularly in the GLS1i sensitive A549 cell lines. The initially observed effects of GLS1i treatment were very different to what we expected (Table 1): GLS1i treatment did not affect cell numbers in either cell line when compared to control treatment (Supplementary Figure S2a). Equally unexpectedly, the amount of glutamine consumed was reduced to a much greater extent in the resistant cell line than in the sensitive cell line (Fig. 2a). Intracellular glutamine concentrations were raised in treated conditions in both cell lines (Fig. 2b), particularly in the resistant H358 cell lines compared to controls, in agreement with our expectations. However, intracellular glutamate concentrations were reduced in the GLS1i resistant H358 cell lines only (Fig. 2c). The abundance of TCA cycle intermediates, such as alpha-ketoglutarate (α-KG), citrate and fumarate, was unaffected by treatment of A549 cells with GLS1i. Only α-KG was reduced in H358 cells (Supplementary Figure S3).

**Table 1:** Predicted versus observed effects of treatment with the GLS1i inhibitor using the prevalent culture conditions compared with the optimized culture conditions devised in this study.

Predicted effect of GLS1i treatment…	Prevalent culture conditions	Optimized culture conditions
**…in sensitive A549 cells**		
Cell numbers ↓	✖	✔
Glutamine consumption ↓	✔	✔
Glutamate production ↓	✖	✔
Glutamine (intracellular) ↑	✔	✔
Glutamate (intracellular) ↓	✖	✔
**TCA cycle intermediates (intracellular)**		
α-KG ↓	✖	✖
Citrate ↓	✖	✔
Fumarate ↓	✖	✔
**….in resistant H358cells**		
Cell numbers unchanged	✔	✔
Glutamine consumption ↓	✔	✔
Glutamate production ↓	✖	✔
Glutamine (intracellular) ↑	✔	✔
Glutamate (intracellular) ↓	✔	✔
**TCA cycle intermediates (intracellular)**		
α-KG ↓	✔	✔
Citrate ↓	✖	✖
Fumarate ↓	✖	✔

✔: significantly changed as predicted at *p* < 0.05.

✖: not significantly changed as predicted, i.e. *p* > 0.05.

A first clue on what could be responsible for the lack of effect of the metabolic inhibitor on the cell proliferation and the paradoxical effects on metabolism, was the extracellular concentration of glutamine at the end of this assay: this was very close to undetectable levels, suggesting that during the assay cells had been subject to a concentration of glutamine varying between 2 mM and 0 mM. In the absence of glutamine, an inhibitor of glutaminase should perhaps not be expected to have any effect; either directly or due to metabolic rewiring.

### Explanation: The cellular environment is uncontrolled

**Figure 3:**
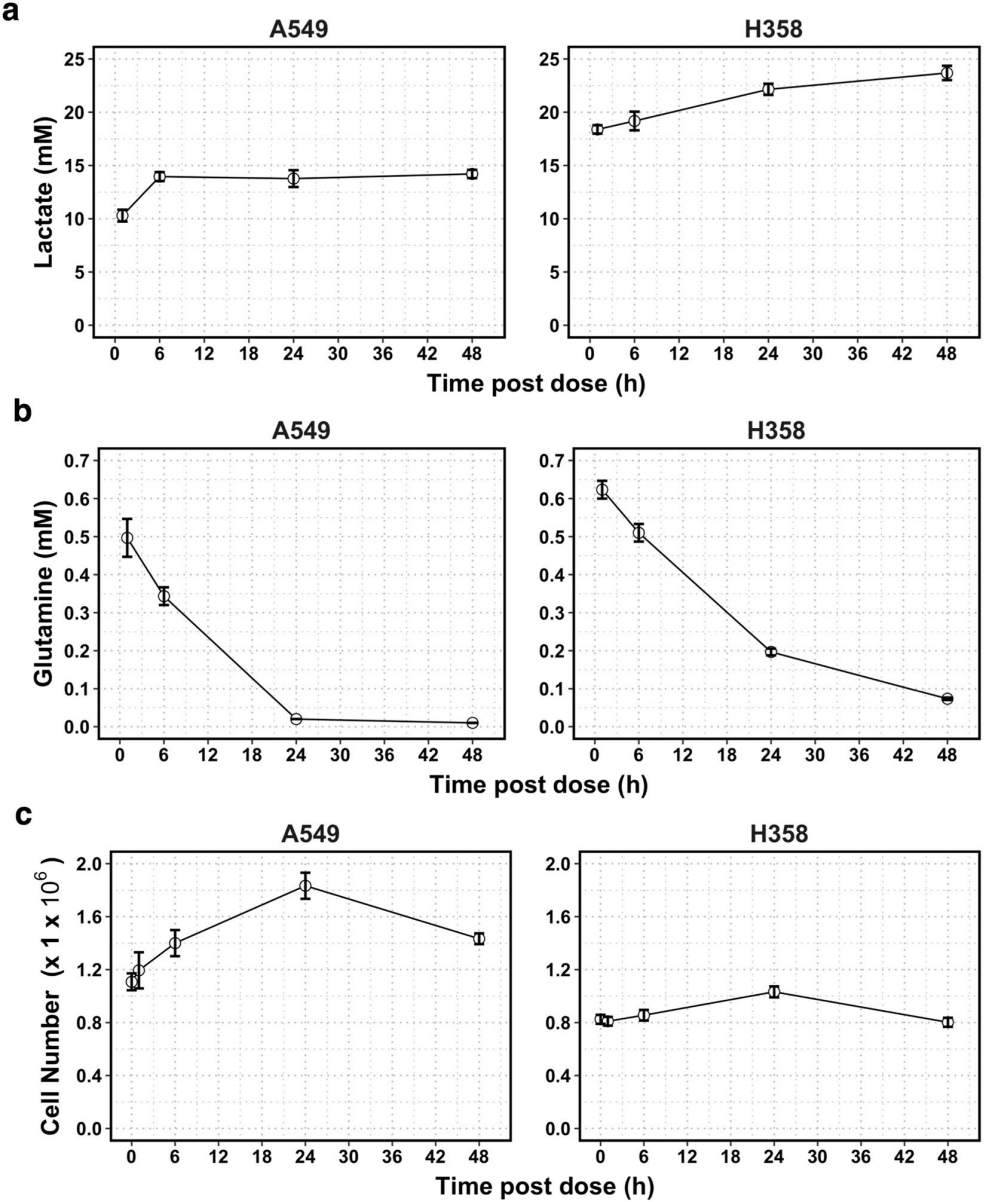
Changes with time after seeding of the state of a cell culture in a traditional assay in vehicle control (0.01% DMSO) conditions. (**a**) Lactate (measured by LC-MS) and (**b**) glutamine (measured by LC-UV) in spent medium. (**c**) Number of live cells per well as measured using the Trypan blue exclusion technique using a Countess automated cell counter (Thermo Scientific, Loughborough, UK). Zero time corresponds to 24 hours after seeding of cells into a medium containing 10 mM of glucose, 2 mM of glutamine, in addition to dialyzed fetal calf serum, vitamins and both essential and non-essential amino acids at concentrations well below 1 mM except for arginine (0.95 mM) and glutamine (2 mM), as shown in Table 2 in Materials and Methods. The cell lines were: A549 (left) and H358 (right). For this single experiment, measurements were performed in triplicate. Shown are the mean ± SEM for the 3 technical replicates per cell line in control conditions.

To understand why GLS1i treatment in the above assay failed to show any significant effects on proliferation or metabolism by A549 and H358 cells we examined the changes in cell numbers and intracellular and extracellular metabolites with enhanced time resolution (Fig. 3 and Supplementary Figure S4).

Our results suggest that, throughout the course of the assay, the cells’ environment in control conditions was changing in ways that would be expected to interfere with the cells’ internal state^26^. Firstly, the concentration of lactate in spent media 1 hour into the assay was already above 10 mM in both cell lines (Fig. 3a). H358 cells had already secreted nearly 20 mM of lactate by this point, suggesting that most of the glucose available in culture media had already been consumed. In H358 cells this increase in lactate continued over the time points sampled, but in A549 cells the concentration of lactate reached its maximum level of around 14 mM 6 hours post dosing. Secondly, the concentration of glutamine in spent media was already reduced by ≥70% 1 hour into the assay in both cell lines and undetectable by 24 hours and 48 hours post dose in A549 cells and H358 cells, respectively (Fig. 3b).

The fluctuating environment that the cells were exposed to in this assay likely contributed to the changes in the specific growth rate of these cells: a small increase in cell numbers was observed over the first 24 hours post dosing (Fig. 3c) but was much slower than the expected growth kinetics of these two cell lines^27^. Moreover, between 24 and 48 hours post dosing, the number of cells in control conditions seemingly *decreased*. This could be due to the depletion of glutamine, glucose or other essential substrates not measured, to the increases in the concentration of lactate, to the resulting decrease in pH, or to contact inhibition of the cells. Deprivation of nutrients and growth factors has been shown to lead to cell cycle arrest and subsequently cell death in NSCLC cell lines indicating that this is a possible explanation for the changes seen in cell numbers in this type of assay^28–30^. The drastic reductions in glutamine could influence normal cell metabolism and physiology, as a result of forcing cells to switch to alternative fuel sources and to deal with the problem of ammonium toxicity^31^. Indeed, the intracellular concentration of glutamine fell drastically throughout the assay, as did the abundance of other metabolites, albeit to a smaller extent (Supplementary Figure S4). The high concentration of secreted lactate in spent media is likely to be accompanied by drastic acidification of culture media and cellular damage^32,33^; the culture media had a buffering capacity of around 20 mM/pH unit, whilst some 20 mM of lactic acid may have been produced, a large proportion of which was likely derived from glucose.

Our results suggest that these commonly used assay conditions are unsuitable for comparing inhibitors of molecular targets with each other.

### Assay optimization: Reducing the seeding density and increasing culture volume stabilizes cellular state

**Figure 4:**
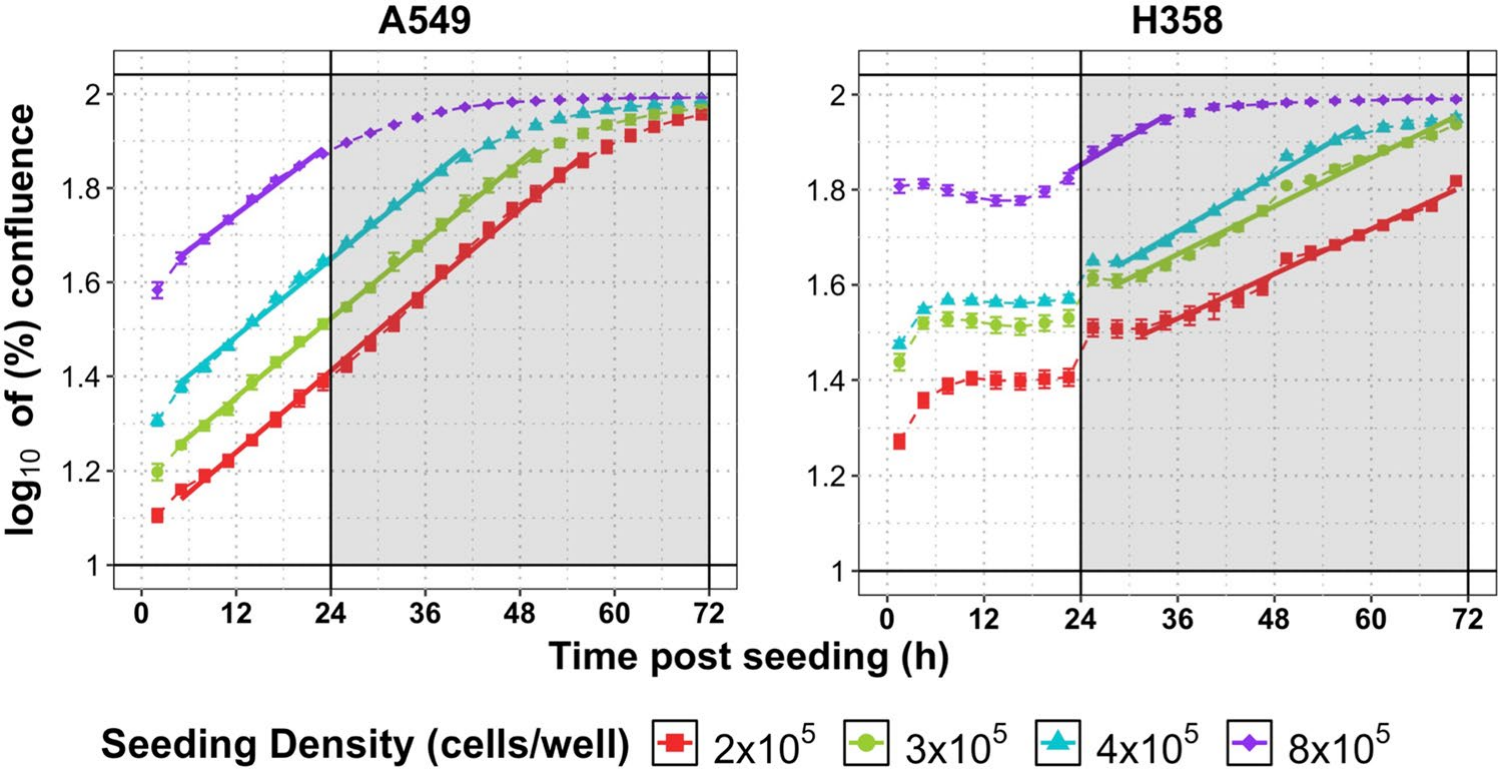
Confluence of A549 and H358 cells over 72 hours after seeding at different initial densities when the volume of culture media was increased to 3 mL. Shown are the changes in log_10_ confluence over time measured by live content cell imaging Incucyte HD system (Essen Bioscience) when A549 and H358 cells were seeded at a density of 8 × 10^5^, 4 × 10^5^, 3 × 10^5^ and 2 × 10^5^ cells/well. For this single experiment, measurements were performed in triplicate for control and treated conditions. Shown are the mean ± SEM for the 3 technical replicates per cell line and treatment condition. Shaded area denotes the assay window in a prevalent assay where samples would be taken over 48 hours from the time of dosing (24 hours after seeding). Solid lines are a fitted linear model for the exponential growth phase of cells.

Increasing the volume of culture media alone from 1 to 3 mL was not sufficient to avoid these problems of variations in metabolic state (Supplementary Figure S5): Whilst this reduced the magnitude of changes in the extracellular concentrations of glucose and glutamine, these key nutrients were still close to depletion 72 hours after seeding. pH changes remained within acceptable ranges in A549 cells, but not in H358 cells where pH changed by >1 pH unit. A slight improvement in the proliferation of these two cell lines was observed but this was still much slower than expected. Confluence was reached early into the assay (24–36 hours after seeding) when the cell lines were seeded at a density of 8 × 10^5^ cells/well (Fig. 4, upper purple line). This, together with the drastic reductions in nutrient concentrations through the assay, may account for the reduced rate of proliferation observed in our previous assays as a result of the induction of cell cycle arrest and apoptosis^8,28–30,34^.

Indeed, we observed steadier metabolic conditions and cell proliferation when the initial seeding density of cells was reduced *and* the volume of culture media increased from 1 to 3 mL (Fig. 4 and Supplementary Figure S5). The period of time during which cells were able to grow exponentially was also increased (Fig. 4). Ensuring that confluence remained below ~80% throughout the assay window (24–72 hours post seeding), or that this level of confluence was reached as late as possible in the assay, required a significant reduction in the initial seeding density of cells, and this was cell-line specific. The time required to recover from reseeding also differed between cell lines and was affected by the initial seeding density. This initial lag phase was very short in duration for A549 cells (<6 hours) compared to the approximately 24 hours required by H358 cells (Fig. 4), which extended beyond 24 hours when H358 cells were seeded at 2 × 10^5^ cells/well. These differences in growth kinetics could well compromise inhibitor assays.

Lowering the initial seeding density of cells also reduced the magnitude of changes in the concentrations of key nutrients such as glucose and glutamine (Supplementary Figure S5b and c), and in pH (Supplementary Figure S5a) throughout the assay window (24–72 hours post seeding). In the case of the H358 cell line, using these conditions, assays beyond 48 hours after seeding may not be suitable since these cells displayed a high rate of glucose consumption (Supplementary Figure S5b) and the corresponding lactate secretion would lead to significant reductions in pH (Supplementary Figure S5a). When H358 cells were seeded at 3 × 10^5^ cells/well, the concentration of glucose reached limiting levels (~2 mM) 72 hours after seeding, which would constitute 48 hours post dosing in an assay where treatment was applied 24 hours after seeding (Supplementary Figure S5b).

We conclude that, in order to ensure that (1) cells are in exponential growth from 24 hours after seeding, (2) confluence is reached as late as possible, and (3) changes in glucose, glutamine and pH are kept to a minimum, the volume of culture media should be increased up to 3 mL and seeding density reduced according to individual cell line growth kinetics. In our case, seeding A549 cells at a density of 2 × 10^5^ cells/well or less, and H358 cells at around 3 × 10^5^ cells/well in 3 mL of culture media, fulfills these criteria.

### Optimized *in vitro* culture conditions enable successful hypothesis validation and discovery

**Figure 5:**
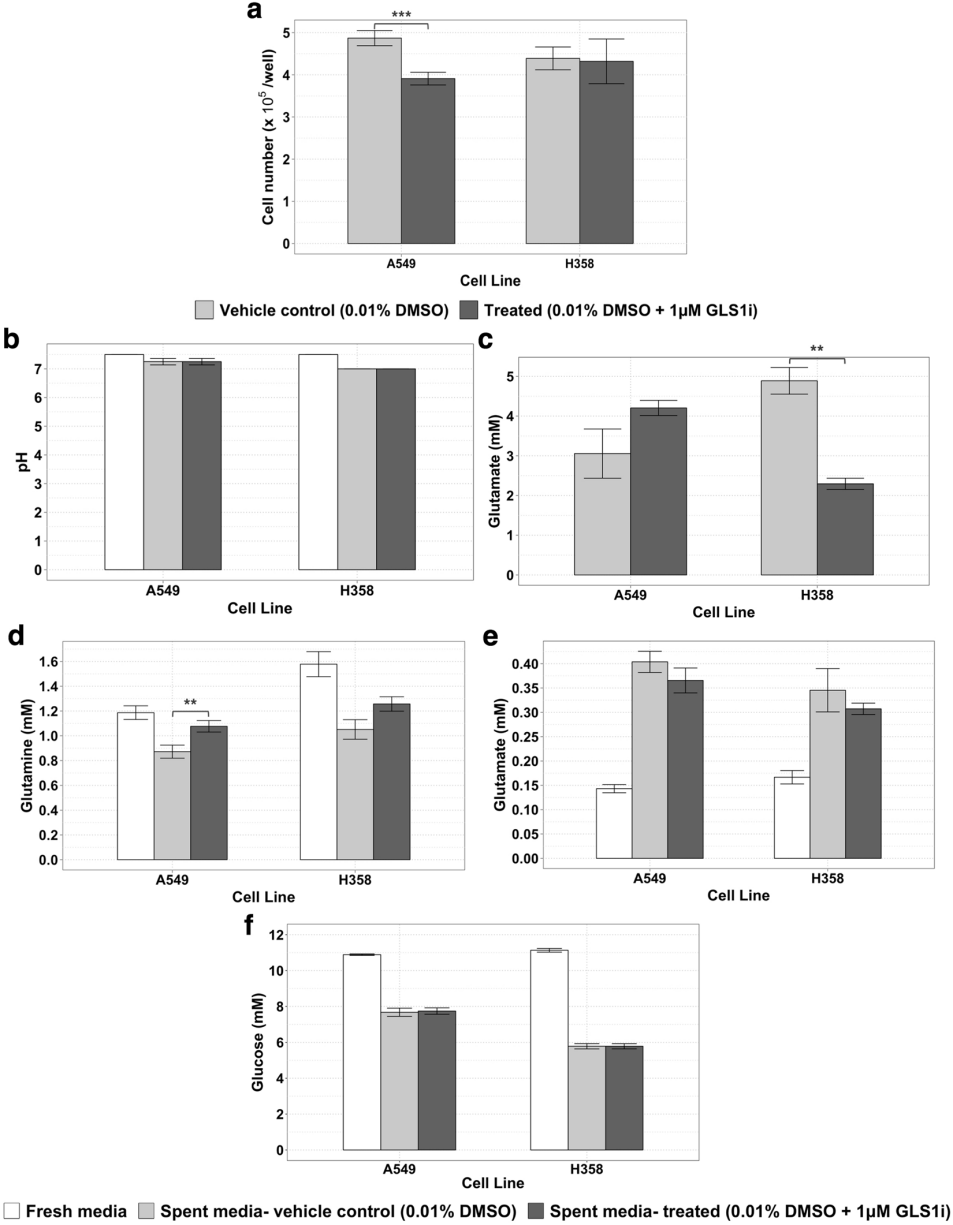
Live cell numbers, concentrations of various metabolites, and pH in fresh and spent media extracts 24 hours after treatment with 0.01% DMSO ± 1.0 μM GLS1i using the improved culture conditions devised here. (**a**) Live cell numbers as measured by automated microscopy following Hoechst staining and fixation (See Materials and Methods). (**b**) pH of fresh and spent media samples measured using MColorpHast indicator strips. Concentration of (**c**) glutamine, (**d**) glutamate, (**e**) glucose and (**f**) lactate in fresh and spent media samples after treatment with or without 1.0 μM GLS1i measured by LC-UV (glutamine and glutamate), Accu-Chek Aviva Blood Glucose Meter System (glucose) and LC-MS (lactate). For each experiment, measurements were performed in triplicate for control and treated conditions. Shown are the mean ± SEM of 3 (A549 cell line; 9 data points per condition) or 2 (H358 cell line; 6 data points per condition) independent experiments. Note that glutamine concentrations in fresh media used for A549 and H358 cells fell by an average of ~27% and ~5% respectively over the duration of the assay. Unadjusted p-values of the differences between control and treated conditions obtained using a two-tailed Student’s t-test are denoted with asterisks: **p ≤ 0.01; ***p ≤ 0.001.

To validate the expected improvement in assay performance we then seeded A549 and H358 cells at a density of 1.5 × 10^5^ and 3 × 10^5^ cells/well respectively in 3 mL of media in a 6 well plate format. Cells were growing exponentially at rates comparable to those reported in the literature^27^ (Supplementary Figure S6) throughout the assay in control conditions. From plates prepared in parallel, the levels of various metabolites in cell and spent media extracts as well as cell numbers were measured for 24 hours after treatment with 1 μM of the GLS1 inhibitor. In agreement with our expectations (Table 1), treatment with the GLS1 inhibitor over 24 hours led to a reduction in cell numbers of around 20% in A549 cells but not in H358 cell lines (Fig. 5a). Throughout the assay, the changes in the cells’ environment were now minimal in both cell lines regardless of treatment conditions (Fig. 5b–f): the concentrations of glucose and glutamine were reduced by less than 50% over the assay and the lactate secreted caused a pH drop <1 unit under these improved assay conditions. The amount of glutamine consumed appeared reduced in both cell lines by treatment with the GLS1 inhibitor although these changes were small and only statistically significant in A549 cells: the achieved stability of culture conditions had the consequence that differences in cellular metabolism were no longer strongly reflected in the changes of the exometabolome, such that assay conditions were now under control and steady.

**Figure 6:**
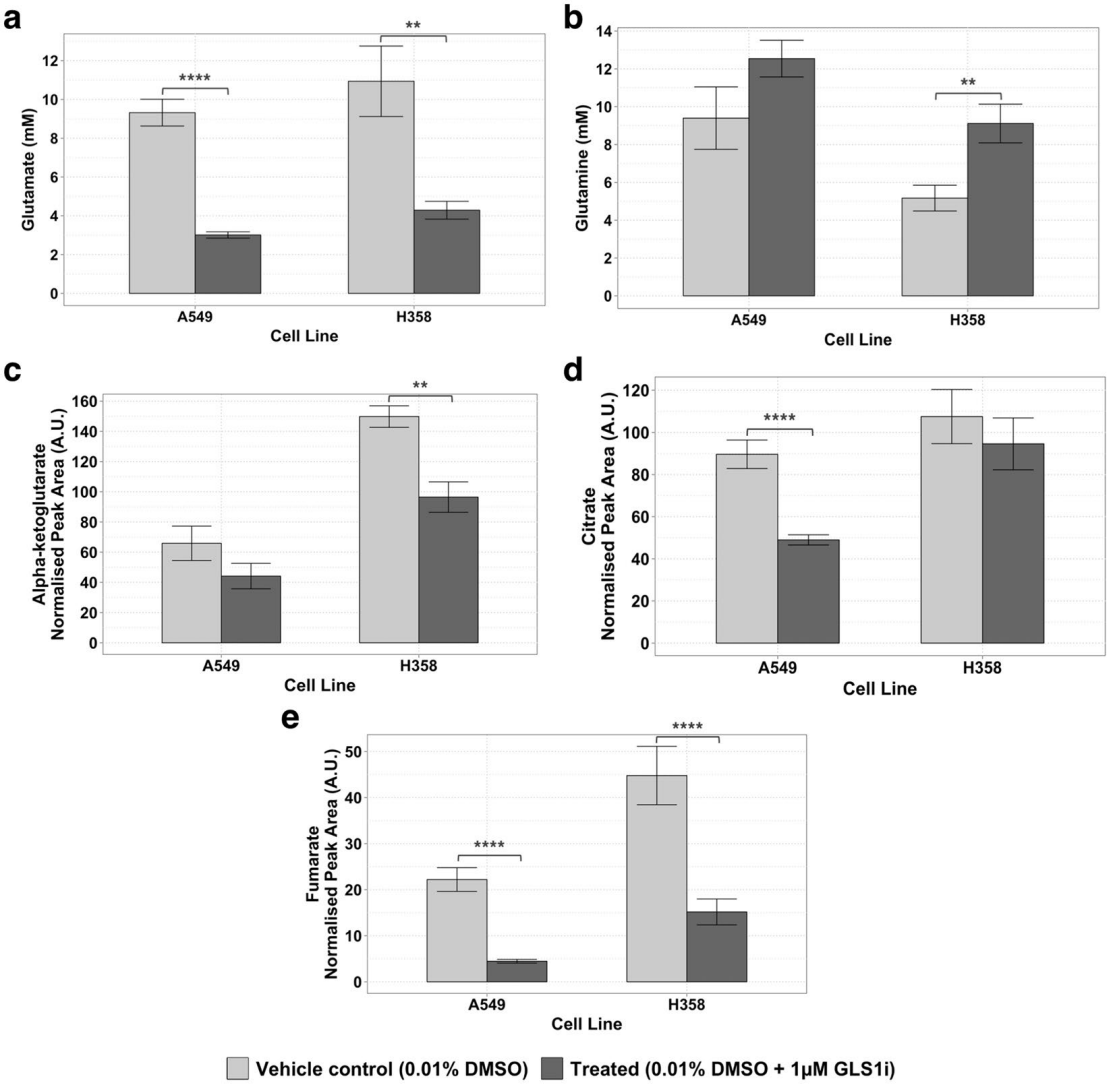
Levels of various intracellular metabolites 24 hours after treatment with 0.01% DMSO ± 1 μM GLS1i using the improved culture conditions devised here. A549 and H358 are known as sensitive and resistant cell lines, respectively. Concentration of (**a**) glutamate and (**b**) glutamine measured by LC-UV, and relative abundance of the TCA cycle intermediates (measured by LC-MS) (**c**) alpha-ketoglutarate, (**d**) citrate and (**e**) fumarate in cells after treatment with or without 1 μM GLS1i. For each experiment, measurements were performed in triplicate for control and treated conditions. Shown are the mean ± SEM of 3 (A549 cell line; 9 data points per condition) or 2 (H358 cell line; 6 data points per condition) independent experiments. Unadjusted p-values of the differences between control and treated conditions obtained using a two-tailed Student’s t-test, the results of which are denoted with asterisks: **p ≤ 0.01; ***p ≤ 0.001; ****p ≤ 0.0001.

We therefore assessed intracellular metabolism to investigate whether, under the optimized conditions, the predicted effects of GLS1i on intracellular metabolites were observed that had not been observed under the previous unstable conditions (Table 1). Our results confirm that the glutaminase inhibitor engaged with the intended target: large reductions (*p* < 0.01) in glutamate were observed in both cell lines (Fig. 6a). Only minor increases in the concentration of glutamine were seen, probably as a result of rapid equilibration with the external medium via the glutamine transporter (Fig. 6b). The intracellular abundance of TCA cycle intermediates was also affected by GLS1i treatment in both cell lines (Fig. 6c–e).

Our results confirm that the optimized culture conditions devised here provide a robust and stable environment in which to reproducibly assay the effects of a GLS1 inhibitor on cell metabolism and proliferation.

## Discussion

The assay with which we started this study failed to demonstrate any consistent effects of glutaminase inhibition on either glutamine metabolism or proliferation in these two cancer cell lines: addition of the inhibitor to cells (A549) known to be sensitive to the inhibitor, had no apparent effect on their proliferation (Supplementary Figure S2a). Conversely, the glutamine metabolism by cells (H358) that are *in*sensitive to the same inhibitor was reduced to a much greater extent than that by sensitive (A549) cells (Fig. 2). We then demonstrated that these inconsistencies were artifacts, for one, because most glutamine had been depleted in the pre-incubation period (Figs 2b and 3b) leaving too little glutamine for effects of the inhibitor to become statistically noteworthy.

With our improved assay conditions we were able to show that the glutaminase inhibitor (GLS1i) does have an effect on glutamine metabolism of both cell lines, but that only the proliferation of the A549 cells is reduced (Fig. 5). For inhibitors of intracellular targets there is always the question of whether these are taken up by cells. Our inhibitor led to an increase in intracellular glutamine and a decrease in intracellular glutamine (Fig. 6a and b), suggesting that the compound entered the cells and affected its target: the glutaminase enzyme.

That the inhibitor used in this paper has been designed so as to be an inhibitor of glutaminase, working at a concentration of 1 micromolar, is not of much importance: it suffices to know that the inhibitor affects cell proliferation. Indeed, we expect that inhibitors of cell proliferation that work through any target should be compromised by the changes in the cells’ environment during the assay, if the conditions are not designed to keep these to a minimum. We used an inhibitor that is of importance to the pharmaceutical industry, because this pinpoints the likely implications of our findings. To be able to do this, we have had to accept that we cannot now disclose the chemical identity of the inhibitor we used.

Our findings highlight the importance of *in vitro* assay optimization for the assessment of the potential of metabolic, and probably also other, inhibitors as anti-cancer drugs that impact on cellular metabolism. Variability in the metabolic state during the assay may well create false positives and false negatives because intermediary and energy metabolism is full of pleiotropic implications. Importantly perhaps, the implications of our findings are unlikely to be limited to studies of metabolic inhibitors. Our observation that cell proliferation assays for drug effectiveness are readily compromised because the metabolic conditions around the cells are changing during these assays should not only impact assays for drugs that act on metabolism. Assays of drugs targeting other aspects of cell biology such as cell division or cell anatomy will also be compromised when cells are dying or changing metabolic state during the assay: changes in metabolic state, e.g. in ATP levels, will affect the dependence of viability on almost any drug. This touches on two important concepts: (i) the various ‘layers’ in cell function are strongly connected, such that one should always consider the other layers when one manipulates only one layer; and (ii) much of the ‘irreproducibility’ of science that is being reported is due not to any experimental mistakes, but due to failure to sufficiently control for these strong interactions, in particular feedback via effects on the wider physiological state of the system under study.

Other inhibitors, such as those of cell signaling or transcription require even longer cell incubations, and may therefore be compromised even more by changes in the levels of metabolites such as ATP, NADH, acetyl-CoA and glutamate that cross-talk widely. Even though metabolism may not be the drug target in many cases, its perturbation due to inappropriate culture conditions, might produce a false response. And since the drug may well affect metabolism indirectly, the impact of metabolic status could be overlooked in both control and treated conditions. Our results suggest that cell assays are even more readily compromised by metabolic changes when the cells are metabolically very active. Naturally, this spells even more trouble for cell types that are relevant for cancer. For robust cell line assays it may be justified that new high-throughput technologies are developed that maintain the extracellular milieu constant over time. Microfluidic technologies may prove suitable for this.

Of course, as highlighted by Haanstra *et al*.^35^ finding drugs that robustly kill cancer cells is not necessarily a good idea. First, all drug actions should be concentration dependent and, second, drugs will also affect normal (non-cancer) cells. In this sense, no drug will kill cancer cells at concentrations that do not affect normal cells^36^. Good drugs are those that kill cancer cells at lower drug concentrations than normal cells. Therefore, assays of anti-cancer drugs need to be both precise and robust.

Perhaps even more so than this, our results should warn against the straightforward implementation of historically-fixed sets of conditions for drug assays in cell lines. Living cells are complex enough to engage in all sorts of metabolic changes, these changes may well differ between individual cell lines, and the metabolome is sensitive to such changes well before the metabolic fluxes produced by the cells are^37^. We therefore advocate that reports on drug assays are accompanied by a thorough description of the experimental procedure used as well as by a metabolic characterization of the cells during the assay, such as in the workflow we demonstrated here. After all, such characterization has become possible over recent years.

Indeed, reports in the literature regarding the characteristics of cell lines under basal and perturbed conditions may have overlooked changes in the metabolic environment of cells or high cell density. Such aspects are typically not reported or measured and may contribute to the irreproducibility of the results when repeating the assay in a different laboratory. Such irreproducibility is fueling the reproducibility debate^1,2^.

Not only do our results highlight the need for reporting experimental ‘details’ concerning culture conditions, they also show a way towards rationalizing and standardizing these. Required details would include, but not be limited to, information on the source of cell lines and passage number (or at least whether all cells used were below a certain passage number), number of cells per well at the time of seeding and throughout the assay, density/confluence throughout the assay, volume of culture media used, details of cell culture flasks used, length of assay (from time of seeding), choice of cell culture medium (including concentrations of all components) and sera (concentration used and source), and how concentrations of key nutrients (e.g. glucose and glutamine) and pH change throughout the assay. This would complement existing efforts for standardization across biomedical research^38–40^ and improve reproducibility, transparency and evaluation of the experimental data, points that are of critical concern^41^.

A number of reporting guidelines for the results of biological assays have been in existence for some time, e.g. the Minimum Information About a Microarray Experiment (MIAME) standard. MIAME is now a reporting requirement for a number of funding agencies and journals^42^. Similarly, there are now minimum reporting standards in use for metabolomics^39^, proteomics^40^ and systems biology models^43^. Since 2008, the Minimum Information for Biological and Biomedical Investigations (MIBBI) project has acted as a repository for the many minimum reporting guidelines that have since been created; there are now over 40 for the biological and biomedical sciences (https://biosharing.org/standards/, accessed 04 July 2016).

The assay developed and discussed here, as well as the contention that it should be accompanied by metabolic analyses of the assay cell lines, should contribute to improved assay reproducibility in cell biology and drug discovery.

## Materials and Methods

### Solvents and Reagents

All solvents used were of HPLC-MS grade and purchased from Sigma-Aldrich (Dorset, UK). Ultrapure water (18.2 MΩ was obtained from a Milli-Q integral water purification system (EMD Millipore, MA, USA) and metabolite standards were purchased from Sigma-Aldrich (Dorset, UK). For cell culture, RPMI-1640 (R7509) medium, Dulbecco’s phosphate buffered saline (PBS, D8537), L-glutamine (G7513) and Penicillin-Streptomycin (P4333) were purchased from Sigma-Aldrich (Dorset, UK). Dialyzed fetal calf serum with SILAC (11698833) was obtained from Thermo Scientific (Loughborough, UK).

### Cell culture

Human lung non-small cell carcinoma cell lines known to be sensitive or resistant to a glutaminase-1 inhibitor (GLS1i) developed jointly by AstraZeneca and Cancer Research Technology (B. Patel and S. Powell, internal communication material; see Supplementary Figure S1) were selected for use in this study. All cells were obtained from the AstraZeneca Cell Bank, which had previously obtained these cell lines from the American Type Culture Collection (ATCC).

Cells were maintained in growth medium consisting of RPMI-1640 minimal media supplemented with 10% (v/v) fetal calf serum and 2.0 mM glutamine in an incubator controlled at 37 °C, 95% humidity and 5% CO_2_. Cells were passaged as required for maintenance when confluence reached around 80% in T175 culture flasks. Cells used in all experiments were below 10 passages.

The composition of the culture media used here is shown in Table 2. RPMI-1640 cell culture media differs from other mammalian cell culture media in that it uses sodium bicarbonate as the buffering system. In addition to around 11 mM glucose, this media also contains vitamins and both essential and non-essential amino acids at concentrations well below 1 mM except for arginine (0.95 mM), but no phenol red. As can be seen in Table 2, the type of RPMI 1640 culture media used here, R7509, does not contain glutamine either, except for the amount we added to a final concentration of 2.0 mM.

**Table 2:** RPMI 1640 (R7509) culture media composition. Source: Sigma-Aldrich.

Component	g/L	MW (g/mol)	mM
**Inorganic Salts**			
Ca(NO_3_)_2_ · 4H_2_O	0.10	236	0.42
MgSO_4_ (anhydrous)	0.049	120	0.41
KCl	0.40	75	5.3
NaHCO_3_	2	84	24
NaCl	6	58	103
Na_2_HPO_4_ (Anhydrous)	0.80	142	5.6
**Amino Acids**			
L-Arginine · HCl	0.20	211	0.95
L-Asparagine · H_2_O	0.050	150	0.33
L-Aspartic Acid	0.020	133	0.15
L-Cystine · 2HCl · H_2_O	0.065	313	0.21
L-Glutamic Acid	0.020	147	0.14
L-Glutamine	—	145	—
Glycine	0.010	75	0.13
L-Histidine · HCl · H_2_O	0.015	155	0.10
Hydroxy-L-Proline	0.02	131	0.15
L-Isoleucine	0.05	131	0.38
L-Leucine	0.05	131	0.38
L-Lysine · HCl	0.04	182	0.22
L-Methionine	0.015	149	0.10
L-Phenylalanine	0.015	165	0.09
L-Proline	0.02	115	0.17
L-Serine	0.03	105	0.29
L-Threonine	0.02	119	0.17
L-Tryptophan	0.005	204	0.02
L-Tyrosine · 2Na · 2H_2_O	0.02883	263	0.11
L-Valine	0.02	117	0.17
**Vitamins**			
D-Biotin	0.0002	244	0.0008
Choline Chloride	0.003	140	0.02
Folic Acid	0.001	441	0.002
myo-Inositol	0.035	180	0.19
Niacinamide	0.001	122	0.008
p-Aminobenzoic Acid	0.001	137	0.007
D-Pantothenic Acid · ½Ca	0.00025	238	0.001
Pyridoxine · HCl	0.001	206	0.005
Riboflavin	0.0002	376	0.0005
Thiamine · HCl	0.001	337	0.003
Vitamin B12	0.000005	1360	0.000004
**Other**			
D-Glucose	2	180	11.10
Glutathione (reduced)	0.001	307	0.003
HEPES	—	—	—
Phenol Red · Na	—	—	—

### Cell culture conditions for metabolomics analysis

Cells were seeded (at the density specified in the text) in a six well plate format in the specified volume culture media (as specified in the text) consisting of RPMI-1640 minimal media supplemented with 10% (v/v) dialyzed fetal calf serum, 2.0 mM glutamine and 1% (v/v) penicillin/streptomycin (10,000 units penicillin and 10 mg/mL of streptomycin).

Experiments were carried out in a six well plate format with 3 wells used for each culture condition. A sample from each well was drawn which was analyzed technically.

Where treatment was applied, this was performed after cells had been incubated for 24 hours to allow cells to adhere to the bottom of wells. Treatment consisted of 0.01% DMSO for control treatment or 1.0 μM GLS1i and 0.01% DMSO (final concentration in wells). After this, growth was continued typically for another 24–48 hours (as specified in the text), without any medium changes.

One plate was prepared for each time point sampled and a parallel plate was also prepared for each time point for cell number determination. An additional parallel plate was also prepared into which culture media was added to wells. This was performed to quantify the levels of metabolites in fresh culture media and account for any changes in their levels over time, in particular glutamine, since this is known to decompose spontaneously in aqueous solution, in a process accelerated by increasing temperatures such as those used in the cultivation of cells (37 °C)^6,7^.

### Extraction of intracellular and culture media metabolites

Metabolites were extracted from cells and fresh or spent culture media at specified time points as detailed below. Wherever possible, steps were performed on ice.

Metabolites in both spent and fresh culture media were extracted by addition of 400 μL of cold extraction solvent (methanol/acetonitrile 50/50 (% v/v) at −20 °C) to a 100 μL aliquot of media taken from the supernatant of the cell cultures in a pre-cooled 1.5 mL tube. This mixture was incubated for 20 minutes at −20 °C and then centrifuged at 16 000 × g for 5 minutes at 4 °C in a Heraeus Biofuge Fresco Refrigerating Centrifuge (Thermo Scientific, Loughborough, UK) to pellet precipitated proteins and any possible cell debris. The supernatant was transferred to a pre-cooled 1.5 mL tube and stored at −80 °C until sample analysis.

Following the removal of spent culture media with an aspirator, cells were washed once, carefully and quickly (i.e. within a few seconds), with 1 mL of PBS at room temperature whilst maintaining plates on ice. Cellular metabolites were extracted by immediate addition of 400 μL of extraction solvent (acetonitrile/methanol/H_2_O 40/40/20 (% v/v/v) at −20 °C) to cells. After incubation for 20 minutes at −20 °C in extraction solvent, cells were macerated by use of a cell scraper. The contents of each well were transferred to a pre-cooled 1.5 mL tube, centrifuged, the supernatant being transferred to a pre-cooled 1.5 mL tube and stored at −80 °C until sample analysis.

### Cell number measurements using the Trypan blue exclusion technique

Cell numbers shown in Supplementary Figure S2a and Fig. 3c were measured in triplicate using the Trypan blue exclusion technique. Parallel 6-well plates were prepared for each time point to allow measurement of cell numbers using a Countess automated cell counter (Thermo Scientific, Loughborough, UK); a benchtop counter designed to measure cell count and viability (live, dead, and total cells) using the Trypan Blue exclusion technique. Cells were treated in the same manner as those prepared for metabolite extraction. At each time point sampled, spent culture media was first removed with an aspirator and cells were washed once, carefully and quickly (i.e. within a few seconds), with 1 mL of PBS at room temperature. 500 μL of Tryp-LE (Thermo Scientific, Loughborough, UK) was subsequently added to detach cells from the well surface by incubating for 2–5 minutes. Once detached, the cells were resuspended in a further 500 μL of pre-warmed (37 °C) media. Cell counting was performed by mixing 10 μL of sample with 10 μL of 0.4% trypan blue, 5 μL pipetted into a Countess chamber slide and cell count and viability measures performed using the Countess cell counter. Three measurements were made for each well to account for pipetting errors.

### Cell number measurements using Hoechst staining

Cell numbers shown in Fig. 5a were determined by nucleus counting. For each experiment, parallel 6-well plates were prepared for each time point sampled to allow measurement of cell numbers. Cells were treated in the same manner as those used to extract metabolites from cells and spent media. Following the removal of spent culture media and washing once with 1 mL of PBS at room temperature, cells were fixed and stained by addition of a solution consisting of 4% formaldehyde with 0.1% Hoechst 33342 (Thermo Scientific Loughborough, UK) in PBS. Plates were incubated for 30 minutes in an incubator controlled at 37 °C, 95% humidity and 5% CO_2_. After this incubation period, cells were carefully washed three times with PBS and subsequently stored in 5 mL of PBS.

Imaging was performed with an ImageXpress Micro automated microscope (Molecular Devices) using a 4 × objective with laser-based focusing. Image analysis and cell count was performed using the Count Nuclei module of the MetaXpress software application (Molecular Devices). Prior to analysis, images and segmentation of individual cells was checked manually for each plate, and where images were out of focus these were discarded from further analysis. For each well, 45 images were taken, which covered 63% of the well area. The cell count data was then converted to *in-situ* cell numbers (multiplying total cell number per well by 1.37). This assumes that there is no overlap between images and that cells are evenly distributed throughout the well. For the purposes of normalization of metabolomics data to cell number only, where cell numbers for a whole time point and treatment condition were missing these were estimated by using an equation obtained after fitting a linear model to the available data.

### Confluence measurements

Proliferation was measured as a function of increasing confluence (Fig. 3 and Supplementary Figure S6) in a 6-well plate format using a live content cell imaging Incucyte HD system (Essen Bioscience) housed inside a standard tissue culture incubator and maintained at 37 °C, 95% humidity and 5% CO_2_. This instrument provides real-time cellular confluence data based on segmentation of high-definition phase-contrast images. Images were taken at regular intervals with a 10× objective from each independent well.

Cells were treated in the same manner as those used for sample preparation for metabolomics analysis and where treatment was added this was added to corresponding wells at the same time i.e. 24 hours after seeding. Cell confluence data, expressed as the mean percentage confluence ± SEM of three wells in each row of each 6 well plate, was exported from the IncuCyte 2011 software in.txt format.

In Fig. 3 changes in confluence over time were measured in one plate per experimental replicate. Using the optimized conditions, confluence measurements in Supplementary Figure S6 were taken in triplicate plates to account for plate-to-plate variability. Since the data provided by the instrument consisted of the mean percentage confluence ± SEM of three wells in each row of each 6 well plate, in order to merge the data for each of the three plates per experimental replicate, the mean of the three means per experimental replicate was calculated and the pooled population variance used to calculate the standard deviation.

Confluence levels over time in each cell line and experimental replicate were plotted using ggplot2 package in R. Where stated, to ensure that cells were growing exponentially, the confluence data was log_10_ transformed and semi-logarithmic plots produced.

### Glucose and pH measurements of media samples

To measure glucose and pH in media, 1 mL of either fresh or spent media was added to a 1.5 mL tube.

A droplet (7.5 μL) of media was placed on an Accu-Chek Aviva Test Strip and read with an Accu-Chek Aviva Blood Glucose Meter System (Roche Diagnostics, Mannheim, Germany). The linear range of the Accu-Chek Aviva Blood Glucose Meter System from 11 mM downwards had been determined previously and found to be in accordance to manufacturer specifications (0.55–33 mM) although the minimum glucose concentration reliably measured was 0.7 mM.

pH of media was measured by addition of 70 μL of media to a non-bleeding pH 2.0–9.0 indicator strip (MColorpHast, Merck Millipore, Darmstadt, Germany).

### Targeted LC-MS Metabolite Profiling

#### Sample preparation for intracellular metabolic profiling

Analytical samples of cell extracts were prepared by addition of an aliquot of cell extract sample to individual 0.3 mL polypropylene microvials which was dried in a Savant SPD1010 SpeedVac concentrator (Thermo, Milford, MA, USA) without temperature application, resuspended in half of the initial volume of ultrapure water and vortexed. A pooled sample was prepared by mixing equal volumes of each analytical sample in a 1.5 mL Eppendorf tube. Quality control (QC) and conditioning samples were prepared from pooled cell extract samples and transferred to two individual microvials, dried down, resuspended in half of the initial volume of ultrapure water and vortexed. A standard sample was prepared by mixing a pre-prepared solution of 143 metabolite standards each at a final concentration of 5 μM. An aliquot of the standard sample was also spiked into a vial containing an aliquot of the pooled sample that had previously been dried down and resuspended in half of the initial volume of water. To assess background and any carry over, a blank sample was prepared with 300 μL ultrapure water in an individual vial.

Analytical samples of cell extracts obtained using the optimised culture conditions were prepared in the same way as described above except that, after drying analytical samples or pooled QC samples, these were resuspended in a quarter of the original volume.

Prior to analysis, all samples were centrifuged at 2,887 × g for 10 minutes at 4 °C in an Allegra X-13 R centrifuge (Beckman Coulter, High Wycombe, UK) to remove any particulates.

#### Targeted metabolic profiling using LC-MS

Intracellular and conditioned media metabolites were analyzed following chromatographic separation and tandem mass spectrometric detection consisting of an Ultimate 3000RS chromatographic system (Thermo, UK) hyphenated to AB 4000 Q TRAP (AB Sciex, UK) mass spectrometer operating in negative ion mode using a protocol described previously^44^. Metabolites were resolved following a gradient elution profile on an UPLC HSS T3 C_18_ 1.8 μm, 2.1 × 100 mm column under temperature (60 °C) controlled conditions. The binary solvent system consisted of buffer A (H_2_O, 10 mM tributylammonium, 15 mM acetic acid) and buffer B (methanol/ isopropanol 80/20) operated at a flow rate of 400 μL/min with a time schedule of: 0 min, 0% B; 0.5 min, 0% B; 4 min, 5% B; 6 min 5% B; 6.5 min, 20% B, 8.5 min, 20%B; 14 min, 55% B, 15 min, 100% B; 17 min 100% B; 18 min 0% B; 21 min 0% B.

#### LC-MS Data Pre-Processing

The raw spectrometric data was integrated with MultiQuant 2.0.2 (Applied Biosystems/MDS Sciex, Warrington, UK). To ensure correct metabolite identification in samples, the peak shape and retention time for each metabolite was visually compared to that of the analyte in the standard mix and spiked sample injections to ensure the correct peak was selected for integration. This data was exported to Excel for preprocessing and normalization.

In Microsoft Excel, the following criteria were applied to determine the presence of a metabolite in a given condition. A metabolite must have been detected in 2 of 3 biological replicates in analytical samples, and also must have been present in >60% of replicate QC injections. To assess technical reproducibility, the percentage coefficient of variation (% QC CV) was calculated for each metabolite detected using data from the QC injections. Metabolites with a % QC CV greater than 30% were excluded from further analysis.

Individual metabolite peak areas were then normalized by dividing by the mean cell number measured at each time point and treatment condition. Data was subsequently normalized to the log_2_ median fold change of peak intensities between samples as described in^45^.

### Lactate quantification in culture media

For quantitation of lactate concentrations in media samples, extracts were diluted 1:100 (v/v) in ultrapure water with addition of ^13^C_3_-labelled lactate (Sigma-Aldrich, Dorset, UK) at a final concentration of 5 μM. Quality control samples were also prepared to assess technical reproducibility by pooling aliquots of analytical samples.

For lactate quantitation the above solvent system was adjusted to isocratic elution of 20% B (0–3 min) followed by 2 min column washing with 100% B before column was re-equilibrated to initial conditions resulting an overall sample analysis time of 8 min. For lactate analysis cell extracts and media samples were diluted with ultrapure water in ratio of 1/10 and 1/100 (extract to final; volume) respectively. ^13^C_3_-lactate spiked at concentration of 5 μM was used as internal standard to normalize for analytical variability.

### Quantification of amino acid concentrations in cell and culture media extracts

Quantification of amino acids in cell and media samples was performed using the Waters Acquity UPLC H-Class system (Waters, Milford, MA, USA). This method combines pre-column sample derivatisation using 6-aminoquinolyl-N-hydroxysuccinimidyl carbamate (AQC), ultra performance liquid chromatography for chromatographic separation and subsequent UV detection.

The 18 individual amino acid standards including histidine (His), serine (Ser), arginine (Arg), glycine (Gly), aspartate (Asp), glutamate (Glu), threonine (The), alanine (Ala), proline (Pro), cystine (Cys), lysine (Lys), tyrosine (Tyr), methionine (Met), valine (Val), isoleucine (Ile), leucine (Leu) and phenylalanine (Phe) were purchased from Waters. Glutamine (Gln) standard was purchased from Sigma-Aldrich (Dorset, UK). The standards mixtures were made up into a stock solution containing 0.25 mM of each amino acid except for cystine (0.125 mM).

In the optimized culture conditions, asparagine (Asn) had also been added to the amino acid quantification method used here. A stock solution containing 0.25 mM Asn was used.

#### Sample preparation for amino acid analysis

Sample preparation was performed using the Waters AccQ-Tag™ Ultra Derivatisation Kit according to the manufacturer’s protocol. Firstly, the derivatisation reagent (AccQ-Tag Ultra Reagent powder, AQC) was reconstituted in AccQ-Tag Reagent Diluent (acetonitrile).

For analysis of amino acid concentrations in Fig. 2b and c, Supplementary Figure S2b, Fig. 3b, Supplementary Figure S4a and b, Fig. 5c and d, and Supplementary Figure S5c, 10 μL of cell or media extract samples was mixed with 20 μL of derivatisation reagent and 70 μL of AccQ-Tag Ultra Borate Buffer in a tapered glass vial. The mixture was vortexed twice with a one minute interval standing at room temperature and incubated in a heating block for 10 minutes at 55 °C.

Standard samples for calibration and amino acid quantitation were diluted 1:10 (v/v) by adding 10 μL of Waters standards mixture and glutamine standard to 60 μL of AccQ-Tag Ultra Borate Buffer and 20 μL of derivatisation reagent in a tapered glass vial, vortexed twice with a one minute interval standing at room temperature and incubated in a heating block for 10 minutes at 55 °C.

A reagent blank and a gradient blank were also prepared. The reagent blank is used to test the quality of derivatisation reagent and was made up by adding 80 μL of AccQ-Tag Ultra Borate Buffer to 20 μL of derivatisation reagent in a tapered glass vial, vortexed twice with a one minute interval standing at room temperature and incubated in a heating block for 10 minutes at 55 °C. The gradient blank tests the quality of the mobile phases and sample preparation solvents; this was made up by mixing 80 μL borate buffer with 10 μL of Waters standard and glutamine standard standards.

Since samples of cell extracts prepared using the optimized culture conditions were more dilute than those prepared using the original culture conditions (Fig. 5a and b), the volumes of each component used in the procedure described above were modified slightly. Here, 30 μL of cell extract sample was mixed with 20 μL of derivatisation reagent and 50 μL of AccQ-Tag Ultra Borate Buffer in a tapered glass vial. In addition, the volumes of each reagent used for the preparation of standard samples and the gradient blank were also modified. Standard samples were prepared by addition of 10 μL of Waters standards mix, glutamine standard and asparagine standard to 50 μL of AccQ-Tag Ultra Borate Buffer and 20 μL of derivatisation reagent in a tapered glass vial. The reagent blank was prepared by mixing 70 μL borate buffer with 10 μL of Waters standards mix, glutamine standard and asparagine standard.

#### Amino acid analysis using LC-UV

Prior to sample analysis, the Waters Acquity UPLC H-Class system was conditioned, followed by injection and analysis of reagent and gradient blanks.

Sample analysis was randomized and a standard sample interspersed at regular intervals.

Chromatographic separation was achieved by injection of 1 μL of sample or standard sample using an ACCQ-TAG ULTRA C18 1 μm, 2.1 × 100 mm column at 43 °C using a gradient mobile phase consisting of 100% AccQ-Tag Ultra Eluent A concentrate (A), 90:10 (% v/v) HPLC-grade water: AccQ-Tag Ultra Eluent B (B), 100% HPLC-grade water (C) and 100% AccQ-Tag Ultra Eluent B (D). Gradient conditions are shown in Table 3.

**Table 3:** Chromatographic gradient elution profile for amino acid analysis.

Time (mins)	Flow(mL/min)	A (%)	B (%)	C (%)	D (%)
0.00	0.70	10.00	0.00	90.00	0.00
0.29	0.70	9.90	0.00	90.10	0.00
5.49	0.70	9.00	80.00	11.00	0.00
7.10	0.70	8.00	15.60	57.90	18.50
7.30	0.70	8.00	15.60	57.90	18.50
7.69	0.70	7.80	0.00	70.90	21.30
7.99	0.70	4.00	0.00	36.30	59.70
8.59	0.70	4.00	0.00	36.30	59.70
8.68	0.70	10.00	0.00	90.00	0.00
10.20	0.70	10.00	0.00	90.00	0.00

A Tunable UV (TUV) detector was used to detect sample absorbance at 260 nm in single wavelength mode.

Data acquisition and processing was performed using Waters Empower 2 chromatography software using manufacturer-defined method for protein hydrolysates. Individual amino acid elution times were manually verified in standard samples prior to processing with the manufacturer defined methodology to quantitate derivatised amino acids. Processed data was exported to Excel and converted to *in situ* concentrations. Alanine co-eluted with an unknown compound therefore this amino acid was excluded from further analysis.

#### Preprocessing of cell and media extract amino acid concentration data

For cell extracts, the raw data was converted to the concentration in the cell extract solution (multiplying by 10 for samples obtained using original culture conditions, 100/30 for samples from optimized conditions). This was then multiplied by the volume of extraction solvent (400 μL) and divided by the mean cell number obtained from cell number measurements to give moles per cell. To get the concentration of amino acid per cell, the number of moles per cell was divided by cell volume, assuming cell volume is around 5 pL^46,47^. The intracellular concentrations are therefore approximate; because the cell volume is uncertain and therefore the concentrations are rough estimates, but the changes in them and the comparison between various metabolites should be more precise.

To convert raw amino acid concentrations in media samples to *in situ* concentrations these were multiplied by a factor of 10 to give the concentration of individual amino acids in the media samples in mM units.

### Statistical analysis

Experiments were carried out with three replicates of the cells each within their own well. A sample from each well was drawn which was analyzed technically.

Statistical significance was determined using paired Student’s t-tests assuming equal variance using the R (version 3.1.2) statistical environment. Data were calculated as mean ± SEM for replicates, technical or biological as indicated.

### Data availability

The source data for all Figures and Supplementary Figures have been provided as Supplementary Dataset 1. All other data supporting the findings of this study are available from the corresponding author on reasonable request.

## Electronic supplementary material

Supplementary Figures

Supplementary Dataset 1
