# Supplementary material for: Rational cell culture optimization enhances experimental reproducibility in cancer cells
Source: Sci Rep. 2018 Feb 14;8:3029. doi: 10.1038/s41598-018-21050-4 (PMC5813001; doi:10.1038/s41598-018-21050-4)
Supplement: Supplementary file 1 — Supplementary Figures [file 41598_2018_21050_MOESM1_ESM.pdf]

Rational cell culture optimization enhances experimental reproducibility in cancer cells

Marina Wright Muelas[1,2,6]*, Fernando Ortega[5], Rainer Breitling[6], Claus Bendtsen[2], Hans V. Westerhoff[1,4,6]

[1]Manchester Centre for Integrative Systems Biology and Doctoral Training Centre, Manchester Institute of Biotechnology, University of Manchester, 131 Princess Street, Manchester M1 7DN, UK

[2]Quantitative Biology, Discovery Sciences, AstraZeneca R&D, Cambridge Science Park, Cambridge, Cambs, CB4 0WG, UK

[4]Netherlands Institute for Systems Biology, VU University Amsterdam and University of Amsterdam, The Netherlands

[5]Manchester Pharmacy School, University of Manchester, Stopford Building, Oxford Road, Manchester, M13 9PT, UK

[6]Manchester Institute of Biotechnology, University of Manchester, 131 Princess Street, Manchester M1 7DN, UK

*Corresponding author: Marina Wright Muelas (marina.wrightmuelas@manchester.ac.uk)

Supplementary Figures

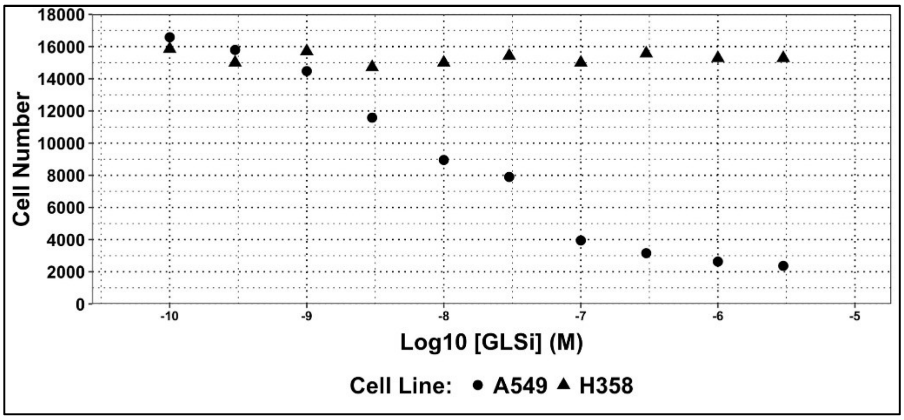

Supplementary Figure S1 Preliminary data showing the effect of the GLS1 inhibitor used in this study on cell numbers in the two cell lines studied here. The effects of a range of concentrations of the GLS1 inhibitor on cell numbers were assessed using a Sytox Green Assay after a 5 day treatment period in A549 and H358 cell lines. Preliminary data, B. Patel and S. Powell, internal communication data.

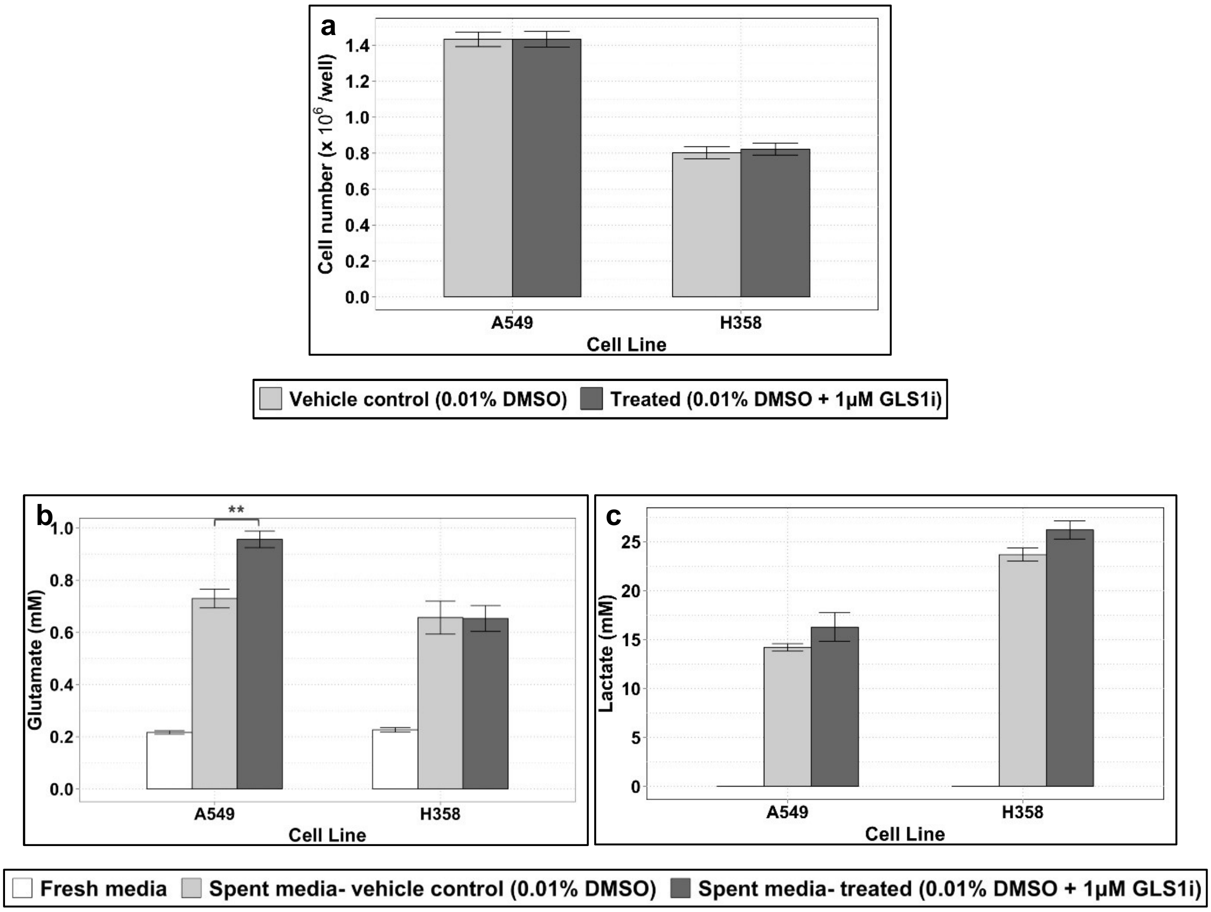

Supplementary Figure S2: Live cell numbers and concentrations of various metabolites in fresh and spent media extracts 48 hours after treatment with 0.01% DMSO ± 1 µM GLS1i using a prevalent assay method. A549 and H358 are known as sensitive and resistant cell lines, respectively. **(a)** Live cell numbers as measured using the Trypan blue exclusion technique using a Countess automated cell counter (Thermo Scientific, Loughborough, UK. Concentration of **(b)** glutamate (measured by LC-UV) and **(c)** lactate (measured by LC-MS) in fresh and spent media samples of cells after treatment with 0.01% DMSO ± 1.0 µM GLS1i. For this single experiment, measurements were performed in triplicate for control and treated conditions. Shown are the mean ± SEM for the 3 technical replicates per cell line and treatment condition. Note that glutamine concentrations in fresh media used for A549 and H358 cells fell by an average of ~30% and ~6% respectively over the duration of the assay. Unadjusted p-values of the differences between control and treated conditions obtained using a two-tailed Student's t-test, are denoted with asterisks: **: $p \leq 0.01$.

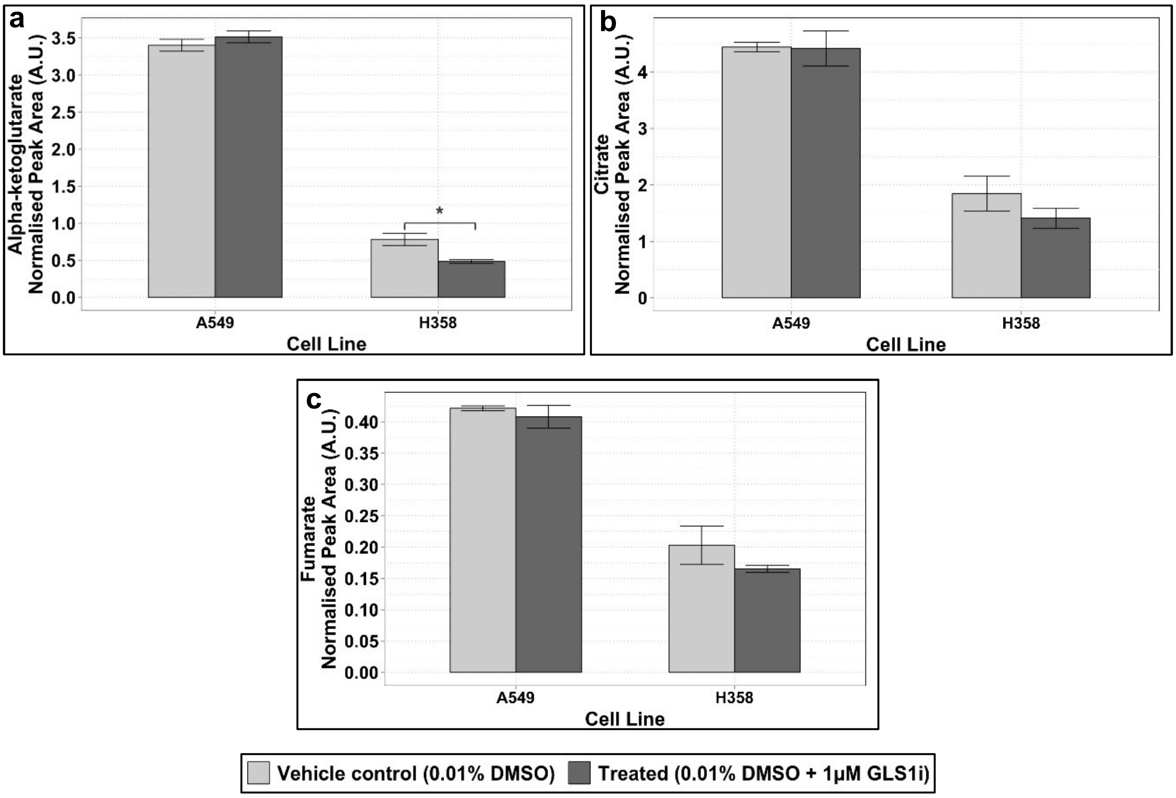

Supplementary Figure S3: Levels of various intracellular metabolites 48 hours after treatment with 0.01% DMSO ± 1.0 μM GLS1i using a prevalent assay method. A549 and H358 are known as sensitive and resistant cell lines, respectively. Normalised peak areas of the TCA cycle intermediates measured by LC-MS **(a)** α-ketoglutarate, **(b)** citrate and **(c)** fumarate after treatment with 0.01% DMSO ± 1 μM GLS1i. For this single experiment, measurements were performed in triplicate for control and treated conditions. Shown are the mean ± SEM for the 3 technical replicates per cell line and treatment condition. Unadjusted p-values of the differences between control and treated samples obtained using a two-tailed Student's t-test, are denoted with asterisks: *: $p \leq 0.05$.

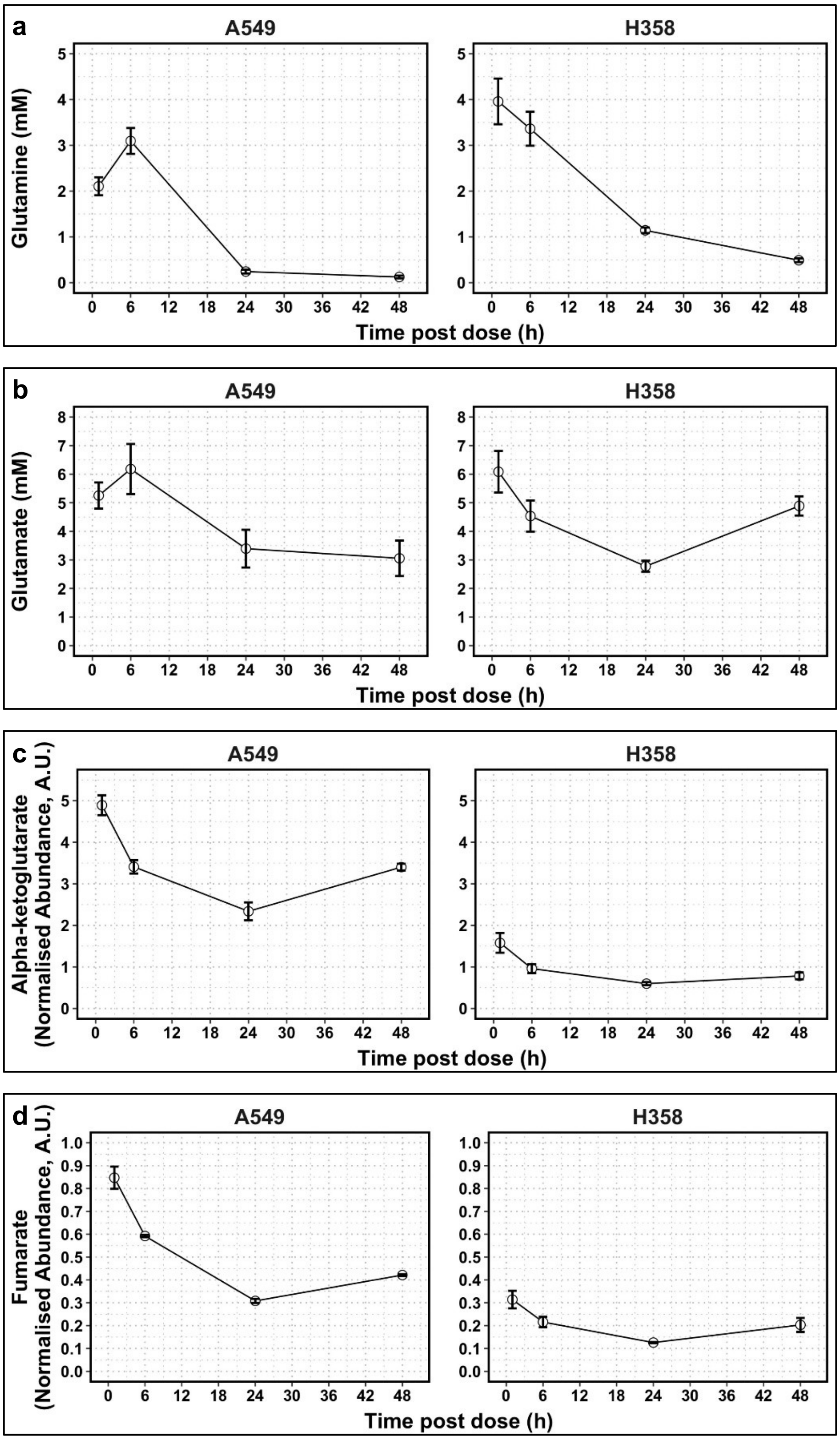

Supplementary Figure S4: Changes with time after seeding in levels of various intracellular metabolites in a traditional assay in vehicle control (0.01%) treatment conditions. Shown are the changes over time of the intracellular concentrations of **(a)** glutamine and **(b)** glutamate as measured by LC-UV, and normalized intracellular abundance of the TCA cycle intermediates measured by LC-MS **(c)** α-ketoglutarate and **(d)** fumarate in a traditional assay. Zero time corresponds to 24 hours after seeding of cells into a medium containing 10mM of glucose, 2 mM

of glutamine, in addition to dialyzed fetal calf serum, vitamins and both essential and non-essential amino acids at concentrations well below 1mM except for arginine (0.95 mM) and glutamine (2.0 mM). The cell lines were: A549 (left) and H358 (right). For this single experiment, measurements were performed in triplicate. Shown are the mean ± SEM for the 3 technical replicates per cell line in control conditions.

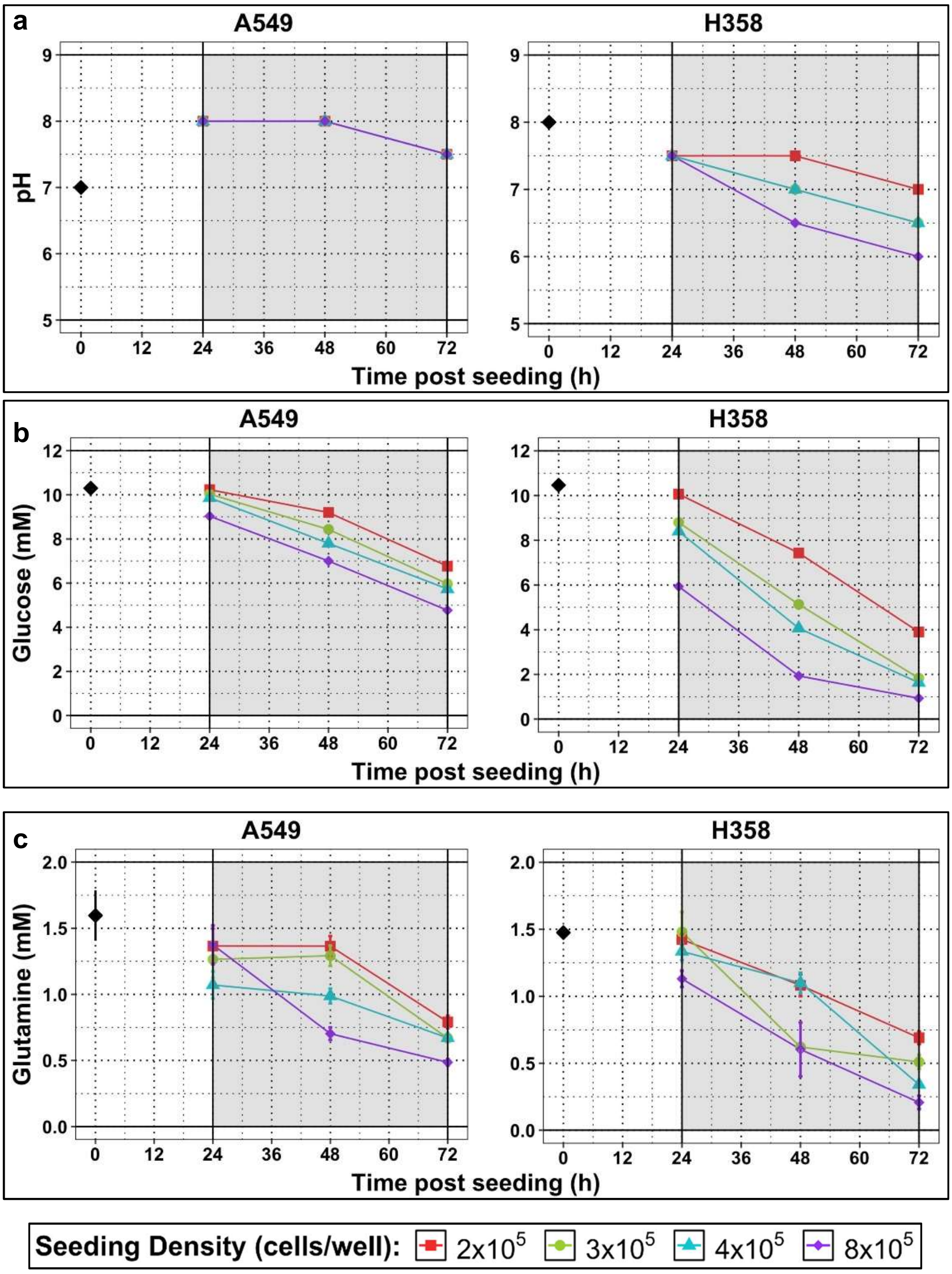

Supplementary Figure S5: Spent media levels of metabolites and pH in A549 and H358 cells over 72 hours after seeding at different initial densities when the volume of culture media was increased to 3 mL. Shown are the changes in **(a)** pH (measured using MColorpHast indicator strips), **(b)** glucose (measured using Accu-Chek Aviva Blood Glucose Meter System) and **(c)** glutamine concentrations (measured by LC-UV) in fresh and spent media over time when A549 and H358 cells were seeded at a density of 8×10^5, 4×10^5, 3×10^5 and 2×10^5 cells/well. For this single experiment, measurements were performed in triplicate for control and treated conditions. Shown are the mean ± SEM for the 3 technical replicates per cell line and treatment condition. The concentration of glucose and glutamine, and pH in fresh media were measured at the time of seeding (t=0h) only (denoted by black diamond). Shaded area denotes the assay window in a prevalent assay where samples would be taken over 48 hours from the time of dosing (24 hours after seeding).

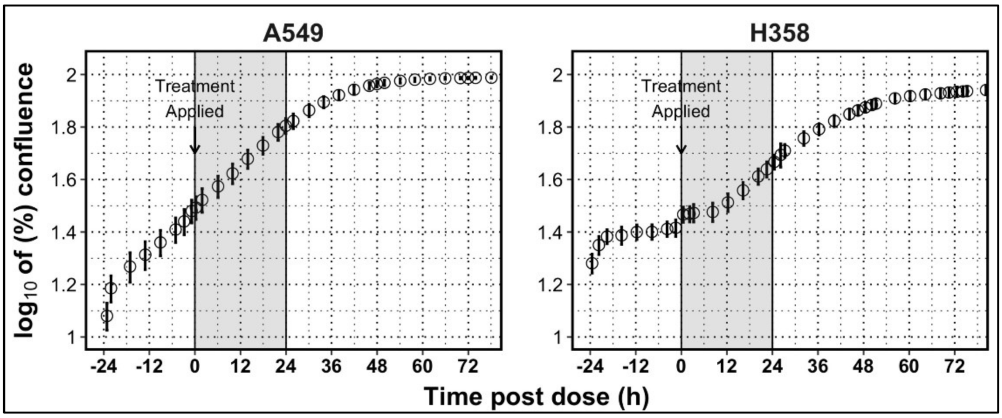

Supplementary Figure S6: Changes in confluence over time in A549 and H358 cells in vehicle control conditions (0.01% DMSO) using the optimized culture conditions devised here. A representative example of confluence changes in each cell line in one experiment are shown as measured by measured by live content cell imaging Incucyte HD system (Essen Bioscience). Confluence was determined in triplicate in three individual 6-well plates. Shaded area denotes the assay window. Shown are the mean confluence ± SEM of samples (9 samples) over a 72 hour period. Note that a logarithmic (log_{10}) scale is used for the ordinate in these plots.
